# Unmasking Cardiovascular Risk in Patients with COPD at Primary Care Settings: The Critical Role of Age, Sex, and Smoking

**DOI:** 10.3390/jcm14051444

**Published:** 2025-02-21

**Authors:** Claire Young, Aaron Courtenay, Kingston Rajiah, Ahmed Abuelhana

**Affiliations:** 1 Mid-Ulster GP Federation, Ballymena BT42 1HL, UK; claire_young2@sky.com; 2 School of Pharmacy and Pharmaceutical Sciences, Ulster University, Coleraine BT52 1SA, UK; a.courtenay@ulster.ac.uk (A.C.); a.abuelhana@ulster.ac.uk (A.A.)

**Keywords:** COPD, cardiovascular comorbidities, primary care, smoking status, general practice

## Abstract

**Background:** Chronic obstructive pulmonary disease (COPD) is a progressive respiratory condition frequently associated with cardiovascular comorbidities, including ischemic heart disease (IHD), heart failure (HF), and atrial fibrillation (AF). These conditions significantly impact patient outcomes, yet their prevalence and risk factors remain underexplored in primary care settings. This study investigates the role of age, sex, and smoking status in the prevalence of IHD, HF, and AF among patients with COPD managed in general practice settings. **Methods:** A retrospective analysis was conducted using de-identified electronic health records from eleven general practitioner (GP) practices in Northern Ireland. Patients with COPD were identified through the Quality and Outcomes Framework (QOF) register, and the presence of IHD, HF, and AF was recorded. Statistical analyses included chi-square tests and independent *t*-tests to examine associations between cardiovascular comorbidities and patient demographics, with significance set at *p* < 0.05. **Results:** Among the total registered population of 77,797, there were 1575 patients with COPD, of whom 335 had IHD, 211 had AF, and 116 had HF. Patients with COPD had a significantly higher prevalence of IHD, AF, and HF compared to those without COPD. Age was a strong determinant, with older age groups, particularly those over 75 years, showing a markedly higher prevalence of all three conditions. Sex analysis revealed that male patients with COPD were significantly more likely to have IHD, AF, and HF than females. However, the association between smoking status and the presence of HF, IHD, or AF was not significantly different between current/ex-smokers and non-smokers. **Conclusions:** The findings highlight the high burden of cardiovascular comorbidities among patients with COPD in primary care and emphasise the need for routine cardiovascular screening. Given the strong influence of age and sex, targeted risk assessment and management strategies should be prioritised for older and male patients with COPD. The lack of association between smoking and cardiovascular comorbidities suggests that additional risk factors should be considered in cardiovascular risk assessment. A more integrated approach to managing COPD and cardiovascular conditions within general practice settings is essential to improve patient outcomes. Future research should focus on evaluating interventions that enhance early detection and multidisciplinary management of cardiovascular comorbidities in patients with COPD.

## 1. Introduction

Chronic obstructive pulmonary disease (COPD) is a diverse lung condition characterised by persistent respiratory symptoms and an exaggerated inflammatory response [1]. Globally, COPD affects an estimated 10.3% of the population, contributing to approximately 3 million deaths annually [2]. This burden is expected to rise due to ongoing exposure to risk factors and an ageing population. In the UK, COPD is the second most prevalent lung disease, with around 2% of the population and 4.5% of individuals over 40 years old living with a diagnosis [3].

In Northern Ireland, COPD is the second leading cause of emergency hospital admissions in the region, with 30% of these patients readmitted within three months and 15% dying within three months of discharge [4]. Patients with COPD often suffer from various comorbidities, including ischemic heart disease, pulmonary hypertension, heart failure, anxiety, depression, malnutrition, and osteoporosis [5]. These extrapulmonary comorbidities negatively impact the quality of life, exacerbate disease progression, and increase mortality, underscoring the need for comprehensive care [6].

Cardiovascular comorbidities are particularly prevalent among patients with COPD, significantly increasing the risk of exacerbations, hospitalisations, and mortality [7]. The prognosis for patients with COPD with cardiovascular disease is worse than the combined prognosis of each condition alone [8]. Patients with COPD face a 2- to 5-fold increased risk of developing cardiovascular disease [9]. Shared risk factors, such as smoking, ageing, and reduced physical activity, contribute to this increased risk [10]. Additionally, the pathophysiological mechanisms of COPD, including hyperinflation, hypoxemia, systemic inflammation, pulmonary hypertension, exacerbations, and oxidative stress, are believed to interact with and exacerbate cardiovascular disease prevalence [11].

Ischemic heart disease (IHD), heart failure (HF), and atrial fibrillation (AF) are among the most significant cardiovascular comorbidities affecting patients with COPD, impacting their overall prognosis [12]. However, these conditions are often underdiagnosed and undertreated in the general COPD population [9]. Attention should be paid to the possibility of co-existence of COPD and the influence of COPD [13]. There are several studies in secondary care settings highlighting that cardiovascular comorbidities affect patients with COPD [10,11,12,13]. However, there is a notable gap in primary care settings especially in general practitioner (GP) practices regarding the prevalence of these comorbidities within patients with COPD. Research is needed to enhance the understanding and detection of these conditions among healthcare professionals, thereby improving patient management and outcomes.

Most of the cardiovascular comorbidities in patients with COPD have been conducted in secondary care settings, where patients often present with more severe disease or are referred for specialist care. Consequently, the incidence and prevalence of IHD, HF, and AF may be higher in secondary care populations. In contrast, primary care settings manage a broader spectrum of COPD severity, including earlier-stage disease, making it important to assess the prevalence in this population. Understanding these differences can help tailor prevention and early intervention strategies.

Comprehensive analysis is a crucial process employed to identify and manage patients with long-term health conditions who are at high risk [14]. A research briefing at the House of Commons Library stated that the prevalence of COPD varies significantly across different geographic regions, with higher rates often found in areas of greater socioeconomic deprivation. Also, unlike many other chronic conditions, COPD has not decreased in prevalence in recent years [15]. In alignment with NHS England’s recommendations, analysis was conducted to support patients with chronic conditions, such as COPD, and to prevent avoidable hospital admissions [16]. This enhances GPs’ ability to provide targeted, direct care to our patients and supports Clinical Commissioning Groups (CCGs) in planning and commissioning health services. For instance, by analysing the number of patients requiring support for COPD, GPs can better manage periods of ill health and improve the overall quality of services provided.

This study aimed to determine the prevalence of IHD, HF, and AF in patients with COPD and examine the role of age, sex, and smoking status in primary care settings. Additionally, the study examined the prevalence of patients with COPD with cardiovascular conditions compared to those without.

## 2. Materials and Methods

### 2.1. Study Site

This study included all twelve GP practices within the Mid-Ulster Federation in Northern Ireland. Following agreement with the Federation Board of Directors, Screen Clinical recruited practices within the Federation by the return of practice agreement forms by email and delivered COPD review clinics in each of the practices involved. Screen Clinical is an independent clinical services provider that collaborates with primary care providers to deliver clinical improvement and medicine optimisation projects. The organisation employs pharmacists to conduct structured reviews, facilitate data collection, and support quality improvement initiatives in general practice settings. In this study, Screen Clinical was responsible for coordinating COPD review clinics, collecting de-identified patient data, and ensuring adherence to best practices in primary care management.

A COPD review clinic is a structured clinical service designed to assess and optimise the management of patients diagnosed with COPD in primary care. These clinics were delivered by pharmacists from Screen Clinical and involved a comprehensive review of patient records, symptom assessment, medication optimisation, and screening for comorbidities such as cardiovascular disease. Patients were identified using the Quality and Outcomes Framework (QOF) register, which flagged individuals with a recorded COPD diagnosis. To participate in the study, each GP was required to complete and return a practice agreement form. This agreement formalised the practice’s consent to take part in the COPD review clinics, allowing Screen Clinical to conduct structured reviews within their setting. The agreement ensured that all participating practices adhered to a standardised protocol for patient identification, data collection, and quality improvement activities.

### 2.2. Study Design

This study was conducted as part of a quality improvement initiative aimed at enhancing the identification and management of cardiovascular comorbidities in patients with COPD. The project involved structured COPD review clinics, data-driven identification of at-risk patients, and recommendations for optimising cardiovascular screening and intervention strategies in primary care settings. While the primary aim was to evaluate cardiovascular risk, the findings also contribute to informing best practices for integrating cardiovascular assessment into routine COPD care.

### 2.3. Data Collection

With permission obtained from all stakeholders, the data from the eleven GP practices were collated as part of this quality improvement project. The data were de-identified, meaning all personally identifiable information was removed, ensuring patient confidentiality while maintaining the ability to analyse relevant clinical data. The researchers obtained data free of charge through secure file transfer using SharePoint.

The total number of patients with chronic conditions in this study was estimated at 28,043. This estimate was derived based on available data from GP practices in Northern Ireland, as an exact count of patients with chronic conditions could not be obtained. The estimate is based on patient records indicating known chronic conditions, accounting for the most common diagnoses such as cardiovascular diseases, respiratory conditions, diabetes, etc. While this number may not reflect an exact count, it provides a reasonable approximation based on the available data. This estimate is about 36% of individuals registered in the eleven GPs (*n* = 77,797); comparable data show 34–40% of adults in the UK have chronic conditions [17].

The QOF register was used to search for patients with COPD having AF, IHD and HF. Patients’ age, sex, and smoking status were recorded. All the data were processed and collated in line with data protection and General Data Protection Regulation (GDPR) legislation. The QOF is a UK-based incentive scheme for general practices, designed to standardise and improve the management of chronic diseases through structured data recording. The QOF register was used in this study to identify patients with COPD and cardiovascular conditions, as it contains coded diagnostic information recorded by GPs during routine consultations. The register ensures consistency in patient identification across different practices, minimising selection bias and ensuring data reliability. Initially, all patients with chronic diseases including cardiovascular conditions, respiratory diseases, diabetes, and other long-term conditions were identified. A subset of these patients who had a confirmed COPD diagnosis was then extracted for further analysis. Cardiovascular conditions, including AF, IHD and HF, were examined within both the COPD and non-COPD groups.

COPD was defined according to the QOF criteria, which require a confirmed diagnosis based on post-bronchodilator spirometry demonstrating airflow obstruction (FEV_1_/FVC ratio < 0.7). Patients were included if they were aged 35 or older, had a recorded COPD diagnosis, and were either current/ex-smokers or non-smokers. Chronic disease, as referenced in this study, broadly refers to long-term conditions such as COPD, cardiovascular diseases, diabetes, and chronic kidney disease. However, the total number of chronic disease patients in this study was estimated due to the lack of an exact count.

GP practice records were systematically searched for diagnoses of IHD, HF, and AF using diagnostic Read/SNOMED codes recorded in the QOF database. IHD was defined as a documented history of myocardial infarction, angina, or coronary artery disease, confirmed by investigations such as ECG, angiography, or stress testing. HF was defined based on a clinical diagnosis supported by echocardiographic evidence of ventricular dysfunction and/or elevated natriuretic peptides (BNP/NT-proBNP). AF was identified through documented ECG-confirmed arrhythmia in patient records. Only coded diagnoses in the GP records were used; unstructured clinical notes were not reviewed.

Patients were classified as having IHD, HF, or AF only if these conditions were explicitly recorded in the GP’s QOF database. The absence of a recorded diagnosis was considered indicative of the absence of the condition, as the QOF framework mandates systematic documentation of chronic diseases. However, given that the underdiagnosis of cardiovascular disease in patients with COPD is well documented, some cases may not have been captured due to variations in diagnostic coding and recording practices.

For analysis, patients were categorised into predefined age groups: 35–44, 45–54, 55–64, 65–74, 75–84, and 85 years and above. These age groups were selected based on standard epidemiological classifications used in COPD and cardiovascular research to allow meaningful comparisons across different life stages.

Missing data were managed using a complete case analysis approach, where patients with incomplete records for key variables (e.g., COPD diagnosis, cardiovascular comorbidities, smoking status, or demographic data) were excluded from specific analyses. Descriptive statistics were used to assess the extent of missing data, and no data imputation techniques were applied. The impact of missing data was considered minimal as all the participating general practices used standardised electronic health records, ensuring a high level of data completeness.

### 2.4. Data Analysis

The data were analysed using MS Excel software (version 2301). The *t*-test was used to compare the means between the groups and the chi-square test for independence was used to measure how expectations compared to the actual observed data. *p* values less than 0.05 were considered statistically significant.

## 3. Results

One of the twelve GP practices within the Federation was in an unexplained crisis at the time of the project and unfortunately did not participate. Out of 77,797 individuals registered in the eleven GP practices, 28,043 had chronic conditions. Among these, 7926 had cardiovascular conditions (1691 with AF, 713 with HF, and 5522 with IHD), and 1575 had COPD. Of the patients with COPD, 662 had at least one cardiovascular condition (211 with AF, 116 with HF, and 335 with IHD). In comparison, 7264 patients with cardiovascular conditions did not have COPD (1480 with AF, 597 with HF, and 5187 with IHD).

Table 1 represents the prevalence of patients with cardiovascular conditions (AF, HF and IHD) without COPD vs. those with COPD. Statistical analysis using a two-sample independent *t*-test revealed significant differences in the prevalence rates of cardiovascular conditions between patients with COPD and those without COPD. For AF, patients with COPD had a significantly higher prevalence (13.39%, SD = 2.37%) compared to patients with no COPD (5.27%, SD = 1.29%) (t = 64.18, *p* < 0.001). This suggests a strong association between COPD and AF among patients with cardiovascular conditions. Similarly, patients with COPD had a higher prevalence of HF (7.36%, SD = 3.13%) compared to patients with no COPD (2.12%, SD = 0.42%) (t = 50.09, *p* < 0.001), indicating that COPD is significantly associated with an increased prevalence of HF. The prevalence of IHD was also higher in patients with COPD (21.26%, SD = 4.08%) than in those without COPD (18.49%, SD = 1.49%) (t = 158.67, *p* < 0.001), indicating that COPD is significantly associated with an increased prevalence of IHD.

Table 2 represents the patients with COPD with and without IHD by age group, sex, and smoking status. The chi-square result indicates a highly significant association between age and the presence of IHD in patients with COPD (χ^2^ = 75.57, *p* < 0.001). The most significant discrepancies were observed in the 75–84 age group, where a higher number of patients with COPD along with IHD was observed than expected. Additionally, there were notable differences across the younger age groups, which contributed to the significant chi-square statistic. These findings suggest that the presence of IHD in patients with COPD varies across different age groups, with older patients showing a higher likelihood of having IHD. This pattern highlights the need for age-specific strategies in managing cardiovascular risk in patients with COPD. An association between sex and the presence of IHD in patients with COPD was also found to be highly significant (χ^2^ = 27.87, *p* < 0.001). Males were more likely to have IHD than females, suggesting that sex may influence the likelihood of IHD in patients with COPD. However, no significant association was found between smoking status and the presence of IHD among patients with COPD (χ^2^ = 2.16, *p* = 0.141), indicating that smoking history did not significantly impact the likelihood of IHD in this population.

Table 3 represents the patients with COPD with and without AF by age group, sex, and smoking status. The chi-square test revealed a highly significant association between age and the presence of AF in patients with COPD (χ^2^ = 114.71, *p* < 0.001). This indicates that the prevalence of AF among patients with COPD increases significantly with age, with the older age groups, especially those aged 85 and above, showing a much higher prevalence of AF compared to younger groups. These results suggest that age is a key factor in the increased risk of AF in patients with COPD. Sex was also significantly associated with the presence of AF in patients with COPD (χ^2^ = 58.42, *p* < 0.001). Males were more likely to have AF compared to females, which may reflect differences in the underlying pathophysiology of both COPD and AF. However, smoking status did not show a significant association with AF presence in patients with COPD (χ^2^ = 3.84, *p* = 0.159), indicating that smoking history did not significantly affect the likelihood of AF in this cohort.

Table 4 represents the patients with COPD with and without HF by age group, sex, and smoking status. The chi-square statistic for the association between age and the presence of HF in patients with COPD was 39.81 with 5 degrees of freedom (*p* < 0.001). This indicates a significant relationship between age and the likelihood of having HF among patients with COPD. Older age groups, particularly those aged 65–84, showed a higher prevalence of HF compared to younger groups, suggesting that age is a key factor in the development of HF in patients with COPD. An association between sex and the presence of HF in patients with COPD was also significant (χ^2^ = 26.79, *p* < 0.001). Males were more likely to have HF compared to females, which is consistent with the known gender differences in both COPD and HF. However, smoking status did not show a statistically significant association with the presence of HF in patients with COPD (χ^2^ = 0.18, *p* = 0.15), indicating that smoking history may not be a critical factor in the development of HF among patients with COPD.

## 4. Discussion

The findings from this study provided compelling evidence of a significant association between chronic obstructive pulmonary disease and the prevalence of cardiovascular conditions. The increased risk of these cardiovascular conditions in patients with COPD compared to all those registered with the GP practices warrants the need to screen all patients with COPD. Specifically, it indicates the need to check for atrial fibrillation, heart failure, and ischemic heart disease, as these three cardiac comorbidities are most commonly occurring in patients with COPD [18]. The data indicated that patients with COPD are substantially more likely to have these cardiovascular conditions compared to their counterparts without COPD, highlighting an important intersection of chronic diseases that deserves further clinical attention. While smoking is not the only cause of COPD, it remains the most common risk factor in Western Europe. However, in this study, a significant number of patients with COPD were non-smokers. This suggests that other contributing factors, such as occupational exposures, environmental pollution, genetic predisposition (e.g., alpha-1 antitrypsin deficiency), and early-life respiratory infections, may play a role in COPD development. These alternative risk factors should be considered when assessing and managing COPD in primary care settings.

The significantly higher mean prevalence rates of AF, HF, and IHD among patients with COPD suggested that COPD may be a critical factor in the exacerbation or co-occurrence of these cardiovascular conditions [19]. For instance, the study revealed a markedly higher mean prevalence of AF among patients with COPD compared to patients with cardiovascular conditions without COPD. This significant disparity might be due to the inflammatory and systemic effects of COPD, which may contribute to the pathophysiology of AF [20]. Also, chronic hypoxia and systemic inflammation, which are hallmarks of COPD, might predispose patients to atrial arrhythmias [21]. This emphasises the importance of vigilant monitoring for cardiac rhythm disturbances in the COPD population. Similarly, the mean prevalence of HF in patients with COPD was found to be significantly elevated compared to those without COPD. This association may reflect the burden that COPD imposes on the cardiovascular system. The increased pulmonary pressures and ventricular load associated with COPD can exacerbate or lead to heart failure [22]. This highlights the necessity for integrated care strategies that address both pulmonary and cardiac function in patients with COPD. The study also found a significant difference in the prevalence of IHD, with patients with COPD showing a markedly higher mean prevalence compared to those without COPD. This significant association suggested that shared risk factors such as smoking [23], systemic inflammation [24], and vascular dysfunction [25] might play a crucial role in the development of IHD among patients with COPD. The substantially higher prevalence of IHD in the COPD group highlights the importance of proactive cardiovascular risk management and regular screening for ischemic heart disease in individuals with COPD.

The analysis of the association between age and cardiovascular comorbidities among patients with COPD provided important insights into how these conditions vary across different age groups. The results revealed a significant association between age and the presence of IHD, AF, and HF in individuals with COPD. This emphasised the crucial impact of age on the prevalence of these cardiovascular conditions in the COPD population. The findings demonstrated a strong link between age and the prevalence of IHD among patients with COPD. The most pronounced differences were observed in the middle-aged to older adult groups, particularly those in the later stages of life [26]. These groups exhibited a significantly higher prevalence of IHD than expected, suggesting that as individuals with COPD grow older, they are at a higher risk for developing ischemic heart conditions. The significance of the findings across younger age groups indicated that while IHD is more common in older individuals, it also affects younger patients with COPD [27], highlighting the need for vigilance across all age ranges. Similarly, the study highlighted a significant association between age and the presence of AF in patients with COPD. The prevalence of AF increases notably with age, with older adults, particularly those in the advanced stages of life, showing a much higher prevalence of AF compared to younger groups [28]. The chronic nature of COPD, characterised by persistent hypoxia and systemic inflammation, may elevate the risk of developing AF in older patients [29]. The association between age and the prevalence of HF in patients with COPD also highlighted the greater risk of heart failure among older populations. The analysis indicates that HF is more common in older age groups, particularly those in their later years. This finding aligned with the understanding that ageing is associated with a decline in cardiac function [30], compounded by the chronic strain on the heart due to COPD-related pulmonary issues [31]. The prolonged pressure on the heart resulting from COPD-induced pulmonary hypertension most likely may contribute to the development of heart failure in these patients [32]. The observed increase in HF prevalence with age highlights the importance of early detection and management of cardiac issues in older patients with COPD to prevent the progression to heart failure.

The analysis of the association between sex and the presence of cardiovascular comorbidities among patients with COPD reveals significant insights into how these conditions are distributed across male and female populations. The findings indicated a notable association between sex and the prevalence of IHD, AF, and HF in individuals with COPD, with males showing a higher likelihood of having these cardiovascular conditions compared to females [33]. The significant association between sex and the prevalence of IHD among patients with COPD highlighted that males are more likely to suffer from ischemic heart conditions than their female counterparts. This could be attributed to factors such as males experiencing higher levels of vascular inflammation and arterial changes that contribute to developing IHD [34]. Hence, there is a need for targeted cardiovascular screening and intervention strategies for male patients with COPD to address the increased risk of IHD. Similarly, the analysis showed a significant association between sex and the prevalence of AF among patients with COPD, with males being more likely to develop AF [35]. Males may have a greater predisposition for atrial enlargement and fibrosis, conditions that are conducive to the development of atrial arrhythmias [36]. This suggests the importance of focusing on AF prevention and management in male patients with COPD. The study also identifies a significant association between sex and the prevalence of HF in patients with COPD, with males being more likely to have heart failure compared to females. This finding can be explained by factors including sex differences in cardiovascular physiology and the impact of COPD on heart function [37]. Males may experience a greater burden of pulmonary hypertension and right ventricular strain due to COPD, which can contribute to the development of HF [38]. This highlights the need for comprehensive care approaches that address the unique cardiovascular risks faced by male patients with COPD, including early detection and treatment of HF.

The exploration of the association between smoking status and the prevalence of cardiovascular comorbidities among patients with COPD reveals important insights into the differential impact of smoking on various heart conditions within this population. The analysis indicated no significant association between smoking status and the presence of IHD among patients with COPD. This suggests that the prevalence of IHD does not differ significantly between smokers (both current and former) and non-smokers within the COPD population. While smoking is a well-established risk factor for both COPD and IHD, the lack of significance in this study could suggest that other factors (such as age, comorbidities, or the severity of COPD) may be playing a more dominant role in the development of IHD among patients with COPD. The overlap of risk factors for both conditions may reduce the specific impact of smoking on IHD prevalence. In this study, there is no significant association between smoking status and the presence of AF among patients with COPD. The association between smoking and AF is inconsistent, particularly in patients with COPD, where factors like frequent exacerbations, left atrial enlargement, and systemic inflammation may dominate smoking’s impact. Additionally, complex interactions involving age, comorbidities, and genetic/environmental factors confound the relationship, making smoking a less significant independent risk factor. These influences highlight the multifactorial nature of AF risk in COPD populations [39,40,41]. Regarding HF, the analysis showed no significant association between smoking status and the presence of HF among patients with COPD. This suggests that the prevalence of HF is similar across both smokers and non-smokers within this group. Though smoking is a known risk factor for HF in the general population, its specific influence may be less prominent in the context of COPD, where the disease itself and its complications play a critical role in the development of HF. This indicates the need for a comprehensive approach to managing heart failure risk that considers a wide range of contributing factors beyond smoking.

### 4.1. Limitations

This study has several limitations that may impact the generalisability and interpretation of the findings. The study population was from a specific geographic area, limiting broader applicability. Additionally, detailed information on comorbidities beyond AF, IHD, and HF, as well as the severity and duration of COPD and cardiovascular conditions, was lacking, which may have influenced the results. Indeed, Obstructive Sleep Apnoea Syndrome (OSAS) through reduced basal and functional capillarity rarefaction, might pose an additional risk in patients at high cardiovascular risk, such as those with COPD [42]. The absence of data on smoking history in terms of pack years and other potential confounding factors, such as diet, physical activity, environmental influences, and medication use, could have affected the associations observed. Variations in diagnostic coding practices across GP records may also have contributed to underreporting or misclassification of cardiovascular conditions. Further research with more detailed data and longitudinal follow-up is needed to address these limitations. This study aimed to examine the prevalence of cardiovascular comorbidities in COPD patients managed in primary care, where a broader spectrum of disease severity exists compared to secondary care. However, without spirometry data or clinical staging information, we could not differentiate between mild, moderate, and severe COPD cases. The inability to stratify by severity may limit the ability to fully understand how disease progression influences cardiovascular risk in this population. Future studies should incorporate COPD severity measures to enhance risk stratification and targeted interventions.

### 4.2. Clinical Implications

The findings from this study highlight several critical areas for clinical practice enhancement. First, there is a need to integrate comprehensive cardiovascular risk assessment and monitoring into the routine care of patients with COPD in GP practices. The significant burden of cardiovascular comorbidities within this population necessitates coordinated care approaches that address both pulmonary and cardiovascular health, thereby improving patient outcomes and quality of life. Given the substantial differences observed across age groups, age-targeted prevention strategies should be prioritised. These strategies include smoking cessation, weight management, and the control of hypertension and cholesterol levels through lifestyle changes and medical interventions. Structured and proactive screening strategies, such as routine ECGs for AF detection and echocardiography for HF assessment, should be incorporated into COPD management pathways to ensure early diagnosis and timely intervention. Standardising screening protocols across GP practices can help identify at-risk patients more consistently and facilitate earlier treatment. Moreover, the study’s results highlight the importance of sex-specific health initiatives. Tailored interventions such as smoking cessation programmes, lifestyle modifications, and specific medical treatments should be developed to address these heightened risks. Personalised care plans focusing on these areas could significantly improve cardiovascular health and outcomes for male patients with COPD, highlighting the necessity for sex-sensitive approaches in clinical practice. Additionally, ensuring equitable access to cardiovascular screening for all patients with COPD, regardless of sex, optimises patient care outcomes. By embedding cardiovascular risk assessment within COPD management strategies, primary care providers can play a crucial role in reducing the burden of cardiovascular disease in this high-risk population.

### 4.3. Future Research

Future research should explore the mechanisms behind the higher prevalence of cardiovascular comorbidities in older and male patients with COPD, focusing on the role of ageing and sex in disease progression. Studies should evaluate the effectiveness of age-specific and sex-specific interventions to reduce cardiovascular risks. Understanding the interplay between ageing, sex, COPD, and cardiovascular health will be crucial in developing tailored care models that address the unique needs of this diverse patient population. This approach is vital for enhancing the quality of life and health outcomes for individuals with COPD, ensuring comprehensive and effective management of their condition.

## 5. Conclusions

Age and sex were key factors influencing the prevalence of cardiovascular comorbidities in patients with COPD. Older age and males were associated with higher risks of IHD, AF, and HF, highlighting the need for age- and sex-specific management strategies. Regular cardiovascular screening is essential for early detection and intervention, particularly for older and male patients with COPD. The lack of a significant difference between smokers and non-smokers suggests that other factors should also be considered in managing cardiovascular health in patients with COPD.

## Figures and Tables

**Table 1:** Prevalence of Cardiovascular Comorbidities in Patients with and without COPD.

Conditions	Number of Patients with Cardiovascular Conditions and Without COPD in 11 GP Practices, *n* = 7264 Mean (SD)	Prevalence Rate Among 28,043 Patients with Chronic Conditions in 11 GP Practices Percentage (SD)	Number of Patients with Cardiovascular Conditions Along with COPD in 11 GP Practices, *n* = 662 Mean (SD)	Prevalence Rate Among 1575 Patients with COPD in 11 GP Practices Percentage (SD)	*p*-Value
Atrial Fibrillation	1480 (23.33)	5.27% (1.29)	211 (6.75)	13.39% (2.37)	0.0004 *
Heart Failure	597 (17.32)	2.12% (0.42)	116 (6.23)	7.36% (3.13)	0.0005 *
Ischaemic Heart Disease	5187 (23.07)	18.49% (1.49)	335 (8.74)	21.26% (4.08)	0.0002 *

* *p* < 0.001.

**Table 2:** Patients with COPD with and without IHD by Age Group, Sex, and Smoking Status.

Characteristics	Number of Patients with COPD and Without IHD (*n* = 1240)			Number of Patients with COPD Along with IHD (*n* = 335)			chi-Square Statistic (χ^2^)	*p*-Value
	Observed	Expected	(O-E)^2^/E	Observed	Expected	(O-E)^2^/E		
Age in years							75.57	0.0005 *
35–44	22	17.32	1.25	0	4.68	4.68		
45–54	115	96.18	3.52	7	25.82	14.50		
55–64	274	251.14	2.03	45	67.86	7.37		
65–74	419	402.92	0.65	93	109.08	2.33		
75–84	309	361.41	7.84	150	97.59	27.12		
85 and above	101	111.04	0.91	40	29.96	3.37		
Sex							27.87	0.0004 *
Male	606	648.89	2.85	219	176.11	10.49		
Female	634	591.11	3.29	116	158.89	11.24		
Smoking status							2.16	0.141
Non-Smoker	203	194.32	0.39	44	52.68	1.43		
Current/Ex-smoker	1037	1045.68	0.07	291	282.32	0.27		

* *p* < 0.001.

**Table 3:** Patients with COPD along with and without AF by Age Group, Sex, and Smoking Status.

Characteristics	Number of Patients with COPD and Without AF (*n* = 1364)			Number of Patients with COPD Along with AF (*n* = 211)			chi-Square Statistic (χ^2^)	*p*-Value
	Observed	Expected	(O-E)^2^/E	Observed	Expected	(O-E)^2^/E		
Age in years							114.71	0.0003 *
35–44	22	19.06	0.45	0	2.94	2.94		
45–54	121	105.70	2.23	1	16.30	14.85		
55–64	308	276.16	3.93	11	42.84	26.16		
65–74	455	443.73	0.29	57	68.27	1.86		
75–84	361	397.92	3.47	98	61.08	23.06		
85 and above	97	122.42	5.25	44	18.58	30.22		
Sex							58.42	0.0002 *
Male	680	715.81	12.68	145	109.19	13.03		
Female	684	648.19	20.70	66	101.81	12.01		
Smoking status							3.84	0.159
Non-Smoker	201	213.94	6.43	46	33.06	7.25		
Current/Ex-smoker	1163	1150.06	1.49	165	177.94	12.85		

* *p* < 0.001.

**Table 4:** Patients with COPD with and without HF by Age Group, Sex, and Smoking Status.

Characteristics	Number of Patients with COPD and Without HF (*n* = 1459)			Number of Patients with COPD Along with HF (*n* = 116)			chi-Square Statistic (χ^2^)	*p*-Value
	Observed	Expected	(O-E)^2^/E	Observed	Expected	(O-E)^2^/E		
Age in years							39.81	0.0004 *
35–44	22	20.40	0.13	0	1.60	1.00		
45–54	120	127.85	0.47	2	14.15	8.61		
55–64	312	324.12	0.36	7	31.88	6.52		
65–74	480	418.57	7.43	32	42.43	2.58		
75–84	403	381.54	0.94	56	52.46	0.20		
85 and above	122	86.52	9.15	19	15.48	2.22		
Sex							26.79	0.0005 *
Male	748	763.40	0.39	77	61.60	17.44		
Female	711	695.60	0.36	39	54.40	8.60		
Smoking status							0.18	0.15
Non-Smoker	228	229.09	0.01	19	17.91	0.12		
Current/Ex-smoker	1231	1229.91	0.03	97	98.09	0.02		

* *p* < 0.001.

## Data Availability

The data presented in the study are stored securely at school of Pharmacy and Pharmaceutical Sciences, Ulster University. Investigators act as custodians for the data processed and generated by the study and they are also responsible for the access to any information included. The data are available upon request from the corresponding author. Due to privacy and institutional regulations, the data are not publicly accessible.
